# Draft Genome Sequence of *Edwardsiella* *piscicida* Strain ACC35.1 Isolated from Diseased Turbot (*Scophthalmus maximus*) in Europe

**DOI:** 10.1128/genomeA.01626-16

**Published:** 2017-02-16

**Authors:** Noemí Buján, Alicia E. Toranzo, Beatriz Magariños

**Affiliations:** Departamento de Microbioloxía y Parasitoloxía, CIBUS-Facultade de Bioloxía and Instituto de Acuicultura, Universidade de Santiago de Compostela, Santiago de Compostela, Spain

## Abstract

*Edwardsiella piscicida* is a bacterial fish pathogen with a high degree of virulence. The strain ACC35.1 was isolated from diseased turbot in Europe. The draft genome sequence comprises 3.84 Mb with a G+C content of 59.8% and >3,450 protein-coding genes.

## GENOME ANNOUNCEMENT

The genus *Edwardsiella*, described in 1965 (1), is composed, at present, of five species: *E. hoshinae*, *E. ictaluri*, *E. tarda*, *E. piscicida*, and *E. anguillarum*. The description of *E. piscicida* resulted from a reclassification of diverse isolates obtained from diseased fish and previously identified as *E. tarda* (2). *E. piscicida* presents a worldwide distribution and has been isolated in a wide range of hosts and ecological niches causing high mortalities (3–6).

Genomic DNA of *E. piscicida* ACC35.1 was extracted using the High Pure PCR Template preparation kit (Roche Diagnostics) following the manufacturer’s instructions. The purified DNA was used to perform a paired-end sequencing run of the library using the 454 GS-FLX system (Life Sciences/Roche). Genome assembly was carried out using Newbler software version 2.7 (Roche Diagnostics). The subsequence assembly produced 139 contigs (>500 bp) resulting in a total genome size of 3,841,631 bp with a G+C% content of 60%. The *N*_50_ contig size was 236,857 bp with the largest contig being 421,015 bp.

Annotation was performed on the Rapid Annotations using Subsystems Technology (RAST) server (7). The genome of isolate ACC35.1 revealed a total of 3,486 coding sequences, 95 tRNAs, eight rRNA operons, and 35 possible missing genes. According to the annotation tool employed, coding sequences for virulence factors and defense genes, such as bacteriocins, adhesion proteins, and several genes involving antibiotic resistance, were found. Moreover, several cell wall components were annotated, highlighting various genes responsible for syntheses of lipid A and implicated in the resistance of polymyxin. Siderophore genes and genes for the mechanisms of iron acquisition and metabolism, as well as phage-coding sequences, such as phage replication and phage tail assembly, were also detected. In addition, 27 coding sequences related to sulfur and aromatic compound metabolism were found.

This genome sequence will be valuable for comparative genomic studies and searches of virulence factors, thus expanding the understanding of this important fish pathogen.

### Accession number(s).

This whole-genome shotgun project has been deposited at DDBJ/EMBL/GenBank under the accession number MPNU00000000. The version described in this paper is the first version, MPNU01000000.
